# Direct IBE fermentation from mandarin orange wastes by combination of *Clostridium cellulovorans* and *Clostridium beijerinckii*

**DOI:** 10.1186/s13568-018-0728-7

**Published:** 2019-01-03

**Authors:** Hisao Tomita, Fumiyoshi Okazaki, Yutaka Tamaru

**Affiliations:** 1 Department of Life Sciences, Graduate School of Bioresources, Mie University, 1577 Kurimamachiya, Tsu, Mie 514-8507 Japan; 2 Department of Bioinformatics, Institute of Advanced Research Center, Mie University, 1577 Kurimamachiya, Tsu, Mie 514-8507 Japan; 3 Research Center of Smart Cell Innovation, Mie University, 1577 Kurimamachiya, Tsu, Mie 514-8507 Japan

**Keywords:** Mandarin orange wastes, Mesophilic Clostridia, IBE fermentation, Consolidated bioprocessing

## Abstract

For a resolution of reducing carbon dioxide emission and increasing food production to respond to the growth of global population, production of biofuels from non-edible biomass is urgently required. Abundant orange wastes, such as peel and strained lees, are produced as by-product of orange juice, which is available non-edible biomass. However, d-limonene included in citrus fruits often inhibits yeast growth and makes the ethanol fermentation difficult. This study demonstrated that isopropanol-butanol-ethanol fermentation ability of *Clostridium beijerinckii* and cellulosic biomass degrading ability of *C. cellulovorans* were cultivated under several concentrations of limonene. As a result, *C. cellulovorans* was able to grow even in the medium containing 0.05% limonene (v/v) and degraded 85% of total sugar from mandarin peel and strained lees without any pretreatments. More interestingly, *C. beijerinckii* produced 0.046 g butanol per 1 g of dried strained lees in the culture supernatant together with *C. cellulovorans*.

## Introduction

Biofuel production from corn and sugar cane has been put to practical use and it has started to realize a low-carbon society using carbon neutral materials. On the other hand, the global population is estimated to reach 9 billion in 2045 from 7 billion (United Nations Department of Economic and Social Affairs, population), and this population growth required to increase food production. Therefore, it is necessary to move on urgently from using food such as corn to non-edible biomass such as agricultural wastes as a raw material for biofuel production. However, such cellulosic biomass is composed of cellulose, hemicellulose, pectin and lignin (Gray et al. 2006). Cellulose is comprised of a linear chain of glucose forming crystalline fibers (Simone and Michael 2015), while hemicellulose consists of not only a monopolymer such as mannan and xylan, but also a heteropolymer such as arabinoxylan, glucuronoxylan, glucomannan, and xyloglucan. In addition, lignin and phenol compounds are assembled with cellulose and hemicellulose. Thus, since the rigid and complex structures are constructed in cellulosic biomass, it is very difficult to degrade them enzymatically.

Some species of Clostridia are known as having ability to degrade cellulosic biomass efficiently using a multiple-enzyme complex called the cellulosome together with non-cellulosomal enzymes (Doi and Kosugi 2004). Among these species, we have been studying on *Clostridium cellulovorans*, which is a mesophilic and anaerobic cellulolytic bacterium (Sleat et al. 1984). *C. cellulovorans* degrades not only cellulose but also hemicellulose and pectin (Tamaru et al. 2010). Other *Clostridium* species are well-known as acetone-butanol-ethanol (ABE) and isopropanol-butanol-ethanol (IBE) fermenters and are employed for a lot of researches from the early 20th century (Jones and Woods 1986). *Clostridium beijerinckii*, which is also a mesophilic and anaerobic bacterium, is known to assimilate monosaccharides such as glucose, xylose, mannose and arabinose, and to ferment organic acids such as acetic acid, lactic acid and butyric acid, and alcohols such as isopropanol, butanol, and ethanol by utilizing various saccharides (Ezeji et al. 2007, Lee et al. 2008).

Orange juice is one of the major fruit juices and is produced 1.6 million metric tons a year (USDA Citrus: World Markets and Trade). Almost same amount of orange wastes as the orange juice comes out as by-product in the orange juice factory. Therefore, it has been considered that such orange wastes are available for non-edible biomass in all over the world. Some parts of orange wastes are used as animal feed, but a large proportion of them has to be disposed of due to high drying and transportation costs (Tripodo et al. 2004). Since much sugars still remain in peel and strained lees of orange wastes, ethanol fermentation by *Saccharomyces cerevisiae* has been studied. However, d-limonene, hereafter called limonene, which is included in citrus oranges, had extremely toxic effect to such fermenting microorganisms (Grohmann et al. 1994, Winniczuk and Parish 1997). Therefore, it was necessary to separate limonene before its cultivation or to protect limonene by encapsulation or immobilization (Lane 1983, Pourbafrani et al. 2007). On the other hand, few studies have so far been reported on fermentation from citrus oranges by *Clostridium* species.

In order to effectively use orange wastes, this study demonstrated tolerance of *C. beijerinckii* and *C. cellulovorans* against several concentrations of limonene, and evaluated IBE fermentable ability with *C. beijerinckii* and degrading ability with *C. cellulovorans* in the culture medium including mandarin peel and strained lees as sole carbon sources. Since mandarin oranges are very popular in Japan and have limonene as same as other citrus fruits, we focused on mandarin oranges as agricultural and food-processed wastes in this study.

## Materials and methods

### Miroorganism and culture maintenance

*Saccharomyces cerevisiae* BY4741 was used and precultured anaerobically in YPD media with 2.0% glucose (w/v) (Wako, Osaka, Japan) at 30 °C for 72 h without shaking. YPD media was used for one litter of medium: 10 g of yeast extract (Bacto, MD, USA), 20 g of Pepton (Bacto), 20 g of glucose, and adjusted to pH 6.

The preculture and culture medium for *C. cellulovorans* 743B (ATCC 35296) and *C. beijerinckii* NCIMB8052 (ATCC 51743) was partially modified and used [5]. For one litter of medium, it was prepared with 4 g of yeast extract, 1 mg of Resazurin salt, 1 g of l-cysteine HCl, 5 g of NaHCO_3_, 0.45 g of K_2_HPO_4_, 0.45 g of KH_2_PO_4_, 0.3675 g of NH_4_Cl, 0.9 g of NaCl, 0.1575 g of MgCl_2_∙6H_2_O, 0.12 g of CaCl_2_∙2H_2_O, 0.85 mg of MnCl_2_∙4H_2_O, 0.942 mg of CoCl_2_∙6H_2_O, 5.2 mg of Na_2_EDTA, 1.5 mg of FeCl_2_∙4H_2_O, 0.07 mg of ZnCl_2_, 0.1 mg of H_2_BO_3_, 0.017 mg of CuCl_2_∙2H_2_O, 0.024 mg of NiCl_2_∙6H_2_O, 0.036 mg of Na_2_MoO_4_∙2H_2_O, 6.6 mg of FeSO_4_∙7H_2_O, 0.1 g of *p*-aminobenzoic acid), and was adjusted to pH 7 for *C. cellulovorans* and to pH 5 for *C. beijerinckii*, respectively. *C. cellulovorans* and *C. beijerinckii* were anaerobically precultured in the above medium with 0.5% (w/v) cellobiose (Sigma, MO, USA) and with 2.0% (w/v) glucose, respectively, at 37 °C for 23 h without shaking.

### Measurement of total sugar and reducing sugar concentration

Total sugar concentration was measured by phenol–sulfuric acid method. Reducing sugar was measured by DNS method, as d-glucose equivalents.

### Alcohol concentration

Alcohol concentration was measured by a gas chromatograph GC-2010plus (Shimadzu, Kyoto, Japan) with a capillary column Rt-Q-BOND (30 m, inner diameter. 0.32 mm; RESTEK, PA, USA). The oven temperature was 250 °C and the column temperature was 150 °C. Nitrogen was the carrier gas and set at a flow rate of 1.21 mL/min.

### Determination of cell growth

Cell growth was measured by Lumitester PD-30, LuciPac Pen and ATP eliminating enzyme (Kikkoman Biochemifa, Tokyo, Japan). It is known that integrated intracellular ATP concentration correlates with cell growth (Miyake et al. 2016). Cell growth was estimated by measuring ATP concentration of 0.1 mL of cell culture according to the manufacturer’s instruction.

### Preparation of substrates from mandarin

**Fig. 1:**
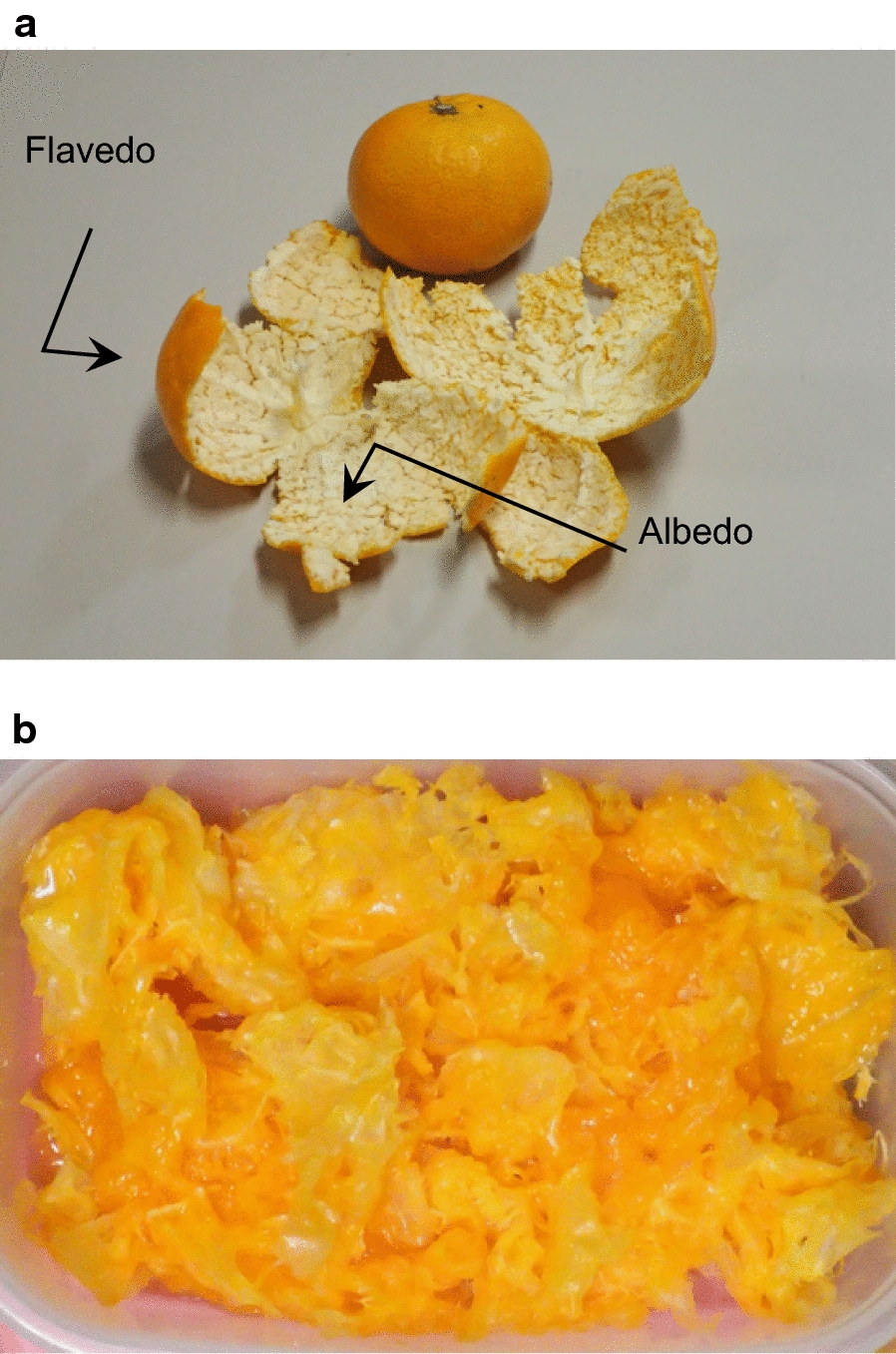
Flavedo and albedo are removed before squeezing (**a**) and after squeezing (strained lees) (**b**)

Mandarin oranges purchased at a grocery store was used. Flavedo and albedo, hereafter called removed peel, were removed before squeezing (Fig. 1a). Whole mandarin oranges except removed peel were squeezed by a squeezing device (Fig. 1b). 10 vials of a medium containing removed peel were prepared. Mandarin oranges were squeezed by SJC-75-W (Irisohyama, Miyagi, Japan).

### Statistics

The data were analyzed for statistical significances using Welch’s *t* test. Difference was assessed with two-side test with an α level of 0.05.

## Results

### Ethanol fermentation and glucose consumption with *S. cerevisiae* under different concentrations of limonene

**Fig. 2:**
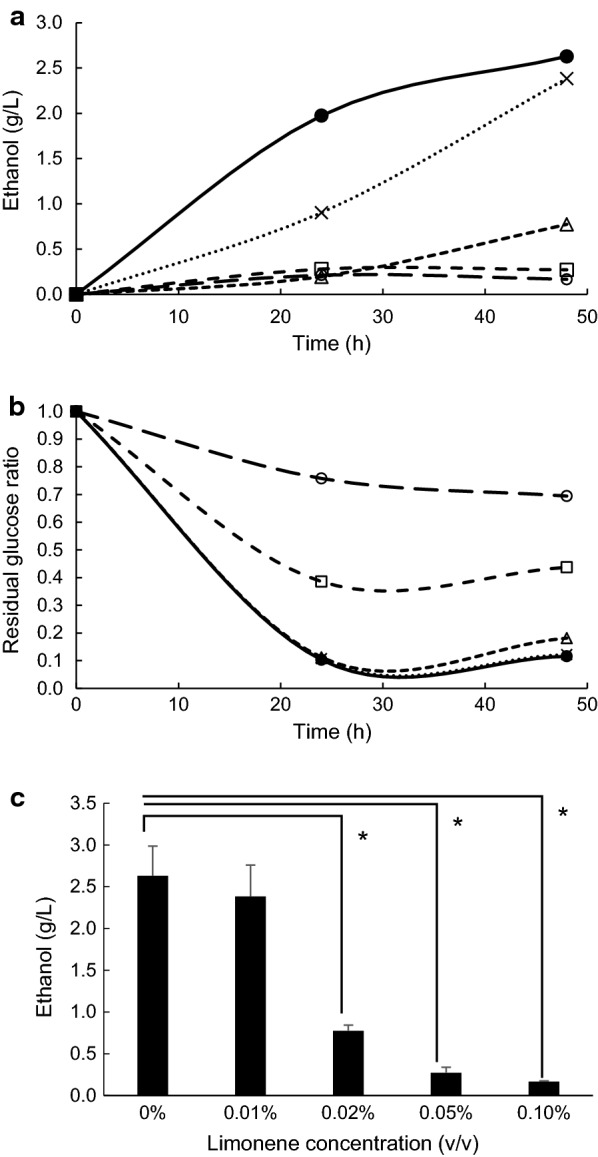
Concentration of ethanol (**a**) and residual glucose ratio (**b**) in the culture medium with *S. cerevisiae*, where different concentrations of limonene (v/v), 0% (filled circle), 0.01% (×), 0.02% (open triangle), 0.05% (open square), and 0.1% (open circle), were present in the culture medium. **c** Ethanol production at 48-h cultivation. Values are mean ± SE of three independent samples. An asterisk indicates a significant difference (p < 0.05)

Anaerobic batch cultivations of *S. cerevisiae* were carried out in a 30-mL YPD medium containing 2% glucose with 0, 0.01, 0.02, 0.05 and 0.1% limonene at 30 °C without shaking. Concentrations of ethanol and glucose were measured at 24- and 48-h cultivations, respectively. Whereas ethanol fermentation was inhibited under more than 0.02% limonene (Fig. 2a), glucose consumption was increased under up to 0.02% limonene (Fig. 2b). Furthermore, ethanol concentration at 48-h cultivation had significant difference from more than 0.02% limonene (Fig. 2c).

### IBE fermentation and glucose consumption with *C. beijerinckii* under different concentrations of limonene

**Fig. 3:**
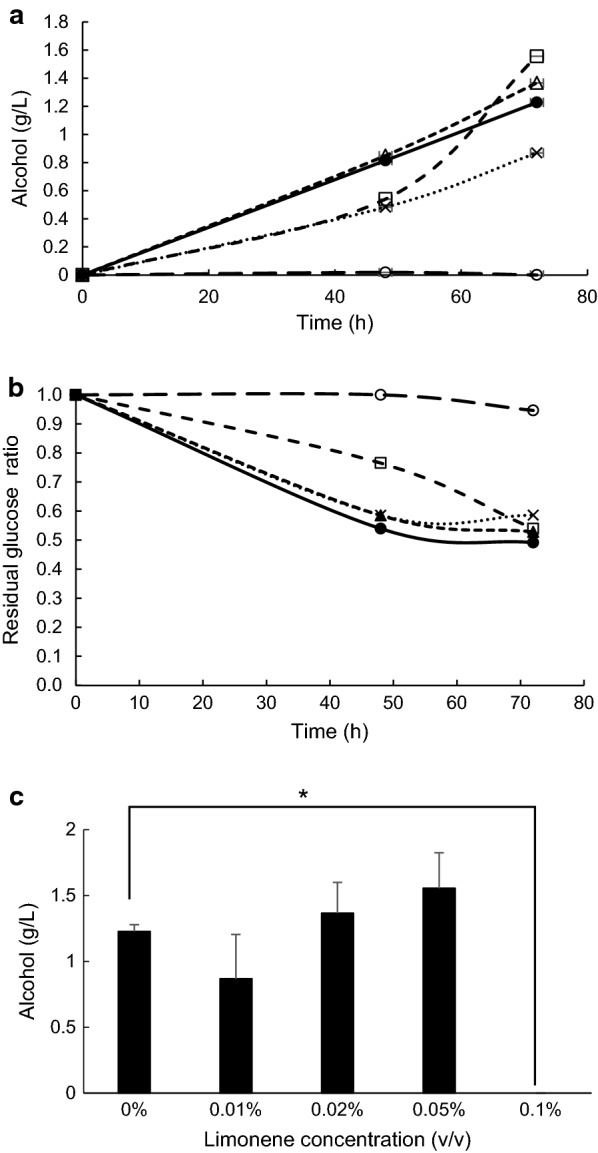
Concentration of alcohol (**a**) and residual glucose ratio (**b**) in the culture medium with *C. beijerinckii*, where different concentrations of limonene (v/v), 0% (filled circle), 0.01% (×), 0.02% (open triangle), 0.05% (open square), and 0.1% (open circle), was present in the culture medium. **c** Alcohol production at 72-h cultivation. Values are mean ± SE of three independent samples. An asterisk indicates a significant difference (p < 0.05)

Anaerobic batch cultivations of *C. beijerinckii* were carried out in a 30-mL medium containing 2% glucose with 0, 0.01, 0.02, 0.05 and 0.1% limonene at 37 °C without shaking. Alcohol and glucose concentrations were measured at 48- and 72-h cultivations, respectively. Total values of ethanol, isopropanol and butanol concentrations were taken as alcohol concentrations. Alcohol production was decreased on 0.05% limonene at 48-h cultivation, but was finally increased at 72-h cultivation (Fig. 3a). On the other hand, glucose consumption showed a similar pattern and reached to about 50% decrease of initial glucose concentration except 0.1% limonene (Fig. 3b). In comparison under several limonene concentrations at 72-h cultivation, alcohol fermentation by *C. beijerinckii* was completely inhibited under 0.1% limonene (Fig. 3c). These results indicated *C. beijerinckii* could ferment glucose to alcohol under less than 0.05% limonene and limonene tolerance of *C. beijerinckii* was five times higher than that of *S. cerevisiae*.

### Cellulose degradation with *C. cellulovorans* under different concentrations of limonene

**Fig. 4:**
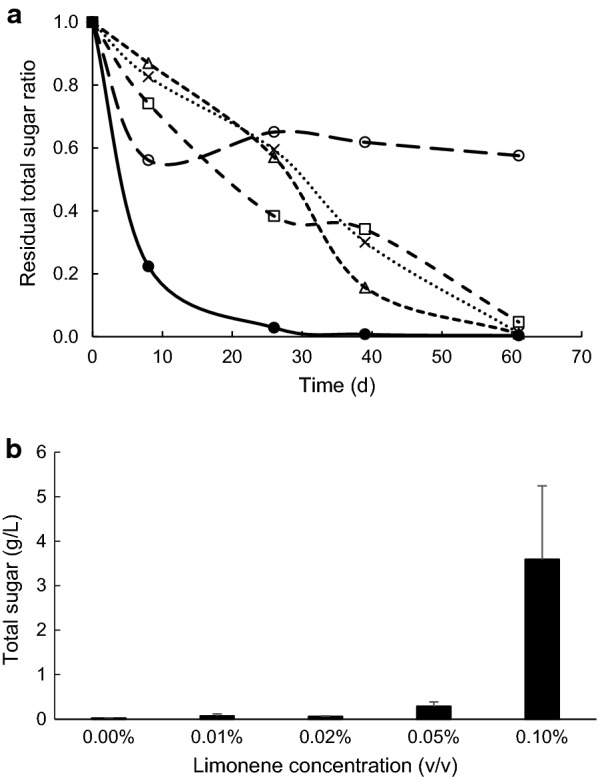
**a** Residual total sugar ratio in the culture medium with *C. cellulovorans*, where different concentrations of limonene (v/v), 0% (filled circle), 0.01% (×), 0.02% (open triangle), 0.05% (open square), and 0.1% (open circle), was present in the culture medium. **b** Total sugar concentration at 61 days cultivation. Values are mean ± SE of three independent samples

Anaerobic batch cultivations of *C. cellulovorans* were carried out in a 30-mL medium containing 0.5% Avicel with 0, 0.01, 0.02, 0.05 and 0.1% limonene at 37 °C without shaking. Total sugar concentrations were measured at 8-, 26-, 39- and 61-days cultivations, respectively. Whereas Avicel was completely degraded done by *C. cellulovorans* without limonene (0%) at 39-days cultivation, approximately 60% degradation was done by it between 0.01 and 0.05% limonene (Fig. 4a). After 61-days cultivation, Avicel was almost completely degraded in the presence of 0.01–0.05% limonene. On the other hand, Avicel was degraded even in 0.1% limonene according to the measurement of total sugar concentration. As a result, there was not a significant difference in comparison with the control (without limonene) (Fig. 4b).

### Degradation of removed peel and strained lees with *C. cellulovorans*

**Fig. 5:**
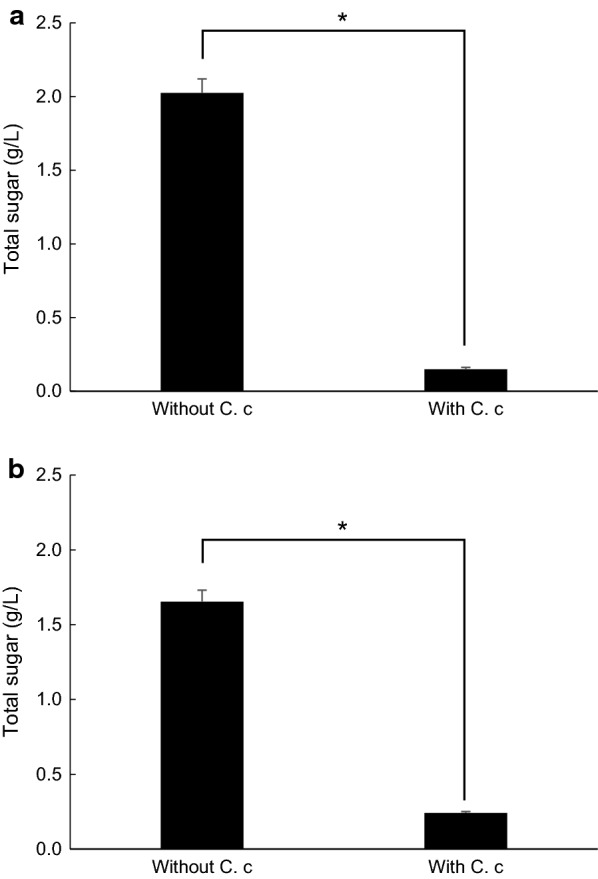
Total sugar concentration in the culture medium containing removed peel (**a**) and strained lees (**b**) degraded with or without *C. cellulovorans*. Values are mean ± SE of three independent samples. An asterisk indicates a significant difference (p < 0.05)

The removed peel was put in a 15-mL vial placed on an electronic scale and the weight was measured except tare. Dry weight was calculated from the water content, of which was 71.6%. The removed peel was added into *C. cellulovorans* medium as 1% (w/v) of a dried substrate. Final volume of the medium was approximately 2 mL for each vial. 10 vials of the medium containing strained lees were made similarly, in accordance with 83.9% water content. Each five vials were inoculated with 0.2 mL of preculture medium containing 0.5% cellobiose with *C. cellulovorans* for both removed peel and strained lees media. All vials were cultivated at 37 °C without shaking. The culture supernatant was removed after centrifugation and total sugar of culture residues was measured after 16-days cultivation. Total sugar in the removed peel media with or without *C. cellulovorans* was 0.148 g/L and 2.025 g/L, respectively (Fig. 5a), while total sugar in the strained lees media with or without *C. cellulovorans* was 0.241 g/L and 1.654 g/L, respectively (Fig. 5b). These results indicated *C. cellulovorans* degraded 93% of removed peel and 85% of strained lees, respectively, without any pretreatment of these substrates.

### IBE fermentation by *C. beijerinckii* from the culture supernatant with *C. cellulovorans*

**Fig. 6:**
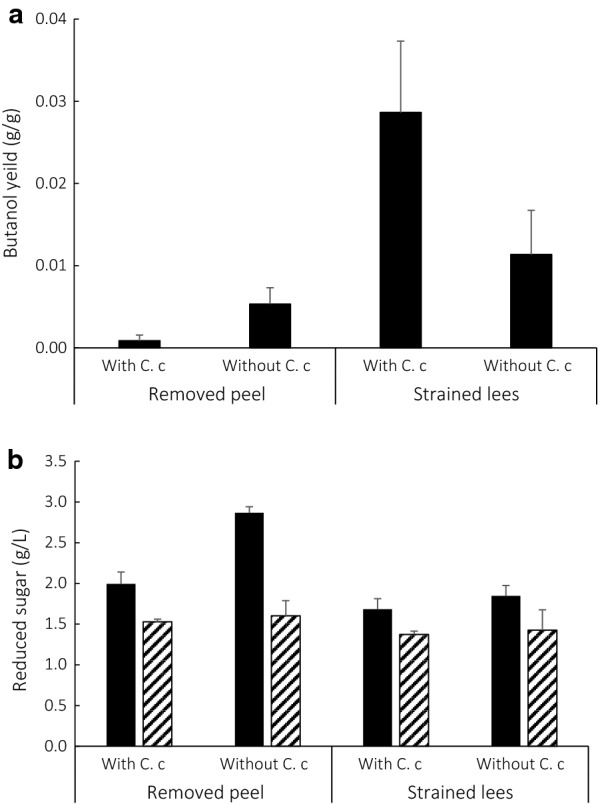
**a** Butanol yield in the culture supernatants with *C. beijerinckii* from removed peel and strained lees with or without *C. cellulovorans*. Values are mean ± SE of four independent samples. **b** Concentration of reducing sugar in the culture supernatant from removed peel and strained lees with or without *C. cellulovorans*. Closed and hatched bars indicate before addition of *C. beijerinckii* and after addition of *C. beijerinckii*, respectively. The cultivation time was for 18 days. Values are mean ± SE of five independent samples

0.1 mL of preculture medium in *C. beijerinckii* was inoculated in 1 mL of culture supernatant after cultivated with *C. cellulovorans* for 16 days, and they were then cultivated at 37 °C without shaking. Butanol concentrations were measured at 18-days cultivation. The measurements of butanol concentration were multiplied by the volume of each vial medium and the weight of butanol per a vial was calculated. The calculated butanol weight was divided by the dry weight of each vial’s substrate as a final yield. Butanol yield from strained lees cultivated with *C. cellulovorans* was twice higher than that without *C. cellulovorans* (Fig. 6a). Namely, maximum yield of butanol was 0.046 g per 1 g of strained lees in the supernatant with *C. cellulovorans*. In contrast, butanol yield was 0.005 g per 1 g of removed peel in the supernatant without *C. cellulovorans*. Moreover, the cultivation conditions were compared with before or after addition of *C. beijerinckii* to the cultivated media with or without *C. cellulovorans*. As a result, reducing sugars in the supernatants after addition of *C. beijerinckii* were always lower than before addition of it (Fig. 6b). In particular, in case of removed peel as a substrate without *C. cellulovorans* and before addition of *C. beijerinckii*, concentration of reducing sugar was highest among all conditions. These results suggested that sugar components for IBE fermentation of *C. beijerinckii* might be different between removed peel and strained lees.

## Discussion

Although the purchase price of cellulosic feedstocks is competitive with petroleum on an energy basis, the cost of lignocellulose conversion to biofuels using today’s technology is high (Lynd et al. 2017). Furthermore, cost reductions can be pursued via either in-paradigm or new-paradigm innovation. In this study, since both *C. beijerinckii* and *C. cellulovorans* are mesophilic anaerobes and grown at 37 °C, it was assumed that consolidated bioprocessing (CBP) between them was synergistically carried out in the same media. It has been reported that *C. cellulovorans* was able to degrade not only cellulose but also corn fibers and plant cell walls such as cultured tobacco and *Arabidopsis thaliana* by formation of their protoplasts (Koukiekolo et al. 2005, Tamaru et al. 2002). Therefore, mandarin orange wastes hit upon a good target for direct IBE fermentation by *C. beijerinckii*. At first, it was demonstrated that tolerance of limonene toxicity against *S. cerevisiae* was measured. Whereas both *C. beijerinckii* and *C. cellulovorans* were cultivated under even 0.05% limonene, *S. cerevisiae* revealed no production of ethanol under over 0.05% limonene (Fig. 2a). In general, it is said that a mandarin orange includes 0.01–0.2% limonene based on season and orange species. In case of *C. cellulovorans*, it degraded 93% of removed peel and 85% of strained lees, respectively (Fig. 5). On the other hand, *C. beijerinckii* produced 0.046 g of butanol per 1 g of strained lees as a dried weight in the culture supernatant with *C. cellulovorans* (Fig. 6a). According to several butanol yields that have been reported in IBE or ABE fermentation by *C. beijerinckii*, butanol (g) per 1 g of glucose was the range within 0.17 to 0.22 g/g (Formanex et al. 1997, Lee et al. 2008, Survase et al. 2011). The reducing sugar concentration in the supernatant of strained lees before *C. beijerinckii* inoculation was 1.68 g/L and butanol concentration from the supernatant was approximately 0.28 g/L. The calculated butanol yield is 0.17 g/g and it is reasonable value compared with the previous reports. These results indicated that there were great advantages to the combination of saccharification and IBE fermentation by mesophilic *C. cellulovorans* and *C. beijerinckii*. Furthermore, *C. cellulovorans* does not require any pretreatment machines, tools or chemicals to degrade mandarin orange wastes. However, this study showed butanol yields by *C. beijerinckii* were different from each part, removed peel and strained lees, of mandarin orange, detail analyses of sugar utilization and its metabolite pathway in *C. beijerinckii* could be feasible and necessary for more studies. In order to optimize the butanol yields by *C. beijerinckii*, it was easier to do for the individual cultivation rather than the co-culture system of *C. cellulovorans* and *C. beijerinckii*. Under the culture conditions optimized for *C. cellulovorans*, orange wastes were quickly degraded and reduced the volume, suggesting that it could be easily recovered by centrifugation. Furthermore, the culture broth would be used as other bacterial sources in the next degradation batch. Likewise, after the centrifugation, the culture supernatant can be optimized for *C. beijerinckii* and the culture broth will be inoculated to the next fermentation batch by cell recycling. In the co-culture system of *C. cellulovorans* and *C. beijerinckii* in a tank, the degradation of orange wastes and IBE fermentation could also be performed, because the degradation and butanol yields varied. It might be necessary to optimize the inoculation ratio of both, but it is difficult to adjust the ratio to inoculate into the next treatment batch from the co-culture broth. Even if it is not the co-culture, it is possible to construct the consolidated process utilizing *C. cellulovorans* and *C. beijerinckii* both without extra enzymes degrading cellulosic biomass. Thus, by degrading orange wastes, the quantity of themselves will be reduced and the costs of drying and transportation on them will be much saved (Sharma et al. 2017). In fact, water contents of removed peel and strained lees were 71.6% and 83.9%, respectively. Furthermore, by consolidated bioprocessing from orange wastes, biobutanol will take the place of fossil fuels such as gasoline and will save energy on the current process.
